# mTOR signaling in cholangiocarcinoma: mechanistic insights and therapeutic opportunities

**DOI:** 10.3389/fphar.2026.1882709

**Published:** 2026-07-15

**Authors:** Zhenxuan Chen, Tao Peng

**Affiliations:** Department of Hepatobiliary Surgery, The First Affiliated Hospital of Yangtze University, The First People’s Hospital of Jingzhou, Jingzhou, China

**Keywords:** autophagy, cholangiocarcinoma, metabolic reprogramming, mTOR signaling pathway, targeted therapy, therapeutic resistance

## Abstract

Cholangiocarcinoma (CCA) is a clinically and molecularly heterogeneous malignancy originating from the biliary epithelium. It represents the second most common primary liver cancer after hepatocellular carcinoma (HCC). Despite advances in understanding CCA pathobiology and improving diagnostic modalities, its global incidence and mortality continue to rise. Most patients are diagnosed at advanced stages, which are characterized by frequent recurrence, metastasis, and therapeutic resistance, leading to poor clinical outcomes. The mammalian target of rapamycin (mTOR) signaling pathway is a central regulator of cell growth, metabolic reprogramming, survival, and autophagy; its dysregulation significantly contributes to CCA initiation and progression. However, the precise roles of mTOR signaling across different CCA subtypes remain incompletely understood, and conflicting evidence exists regarding its context-dependent functions in tumor progression and therapeutic response. This review provides a comprehensive overview of recent advances in mTOR research in CCA, focusing on its involvement in metabolic reprogramming, malignant phenotypes, autophagy, apoptosis, and treatment resistance. Furthermore, current limitations and knowledge gaps in targeting mTOR signaling are discussed, alongside emerging therapeutic strategies, such as mTOR inhibitors, combination approaches, and natural small-molecule modulators. Finally, future research directions and the potential of mTOR-centered interventions to improve clinical outcomes in CCA are highlighted.

## Introduction

Biliary tract cancers encompass a diverse group of malignancies, among which cholangiocarcinoma (CCA) poses a particularly formidable clinical challenge. Arising from the epithelial lining of the bile ducts, CCA ranks as the second most common primary liver cancer after hepatocellular carcinoma (HCC), representing a substantial global health burden (Razumilava and Gores, 2014; Elvevi et al., 2022). Anatomically, CCA is classified into intrahepatic cholangiocarcinoma (iCCA) and extrahepatic cholangiocarcinoma (eCCA). The latter can be further subdivided into perihilar cholangiocarcinoma (pCCA), also known as Klatskin tumor, and distal cholangiocarcinoma (dCCA) (Valle et al., 2016). With improved recognition of CCA and advances in diagnostic approaches, the global incidence and mortality of CCA have continued to increase in recent years (Banales et al., 2026). Multiple risk factors have been implicated in CCA development, including chronic biliary inflammation, cholestasis, primary sclerosing cholangitis, liver fluke infection, hepatolithiasis, and other biliary tract disorders (Yao et al., 2022; Banales et al., 2020). Although advances have been made in surgical resection, systemic chemotherapy, targeted therapy, and immunotherapy, the overall clinical benefit remains limited, particularly in patients with advanced-stage CCA. Poor outcomes are primarily driven by delayed diagnosis, frequent recurrence and metastasis, and the emergence of therapeutic resistance. A better understanding of the molecular mechanisms underlying CCA progression is therefore essential for identifying effective therapeutic targets and improving treatment outcomes (Montal et al., 2020; Liu and Wong, 2026).

The mechanistic target of rapamycin (mTOR) signaling pathway is a central regulatory network that controls cell growth, metabolism, survival, autophagy, and therapeutic response (Zhao et al., 2024). mTOR exerts its biological functions through two distinct multiprotein complexes, mTOR complex 1 (mTORC1) and mTOR complex 2 (mTORC2). These complexes integrate upstream signals from growth factors, nutrient availability, cellular energy status, inflammatory cues, and oncogenic pathways (Yang et al., 2013). In CCA, aberrant activation of mTOR-related signaling pathways, particularly the PI3K/AKT/mTOR and MAPK/ERK cascades, has been closely associated with metabolic reprogramming, dysregulated lipid metabolism, enhanced cell proliferation, epithelial-mesenchymal transition (EMT), tumor invasion, metastasis, and altered therapeutic sensitivity (Carracedo et al., 2008; He et al., 2021; Raggi et al., 2022). These findings suggest that mTOR signaling may serve not only as a key driver of CCA progression but also as a promising therapeutic target.

This review summarizes current evidence regarding the role of mTOR signaling in CCA, with an emphasis on its upstream regulatory mechanisms, downstream biological effects, and therapeutic implications. Specifically, the involvement of key signaling pathways is discussed, including the PI3K/AKT/mTOR and MAPK/ERK axes, as well as metabolic reprogramming, malignant phenotypes, autophagy, apoptosis, and mTOR-targeted therapeutic strategies. By systematically reviewing the biological significance and therapeutic potential of mTOR signaling in CCA, this review aims to provide a comprehensive framework for understanding its role in CCA and to inform the development of more effective molecularly guided therapeutic strategies.

## Literature search strategy

To provide a comprehensive overview of the role of mTOR signaling in cholangiocarcinoma, a structured literature search was conducted to identify relevant preclinical and clinical studies. Major electronic databases, including PubMed/MEDLINE, Web of Science, and Scopus, were systematically searched for articles published up to 2026. The search strategy utilized combinations of the following primary keywords: “cholangiocarcinoma,” “biliary tract cancer,” “mTOR,” “mechanistic target of rapamycin,” “PI3K/AKT/mTOR pathway,” “metabolic reprogramming,” “autophagy,” “chemoresistance,” and “natural compounds.” General inclusion criteria were: (Razumilava and Gores, 2014): peer-reviewed original research articles and authoritative reviews; (Elvevi et al., 2022); studies investigating the molecular mechanisms or therapeutic implications of mTOR signaling specifically in CCA; and (Valle et al., 2016) publications written in English. Conference abstracts, non-peer-reviewed preprints, and studies lacking direct relevance to the mTOR pathway in CCA were excluded. While the search prioritized recent advances published within the last decade, foundational mechanistic studies and seminal clinical trials were also included to provide essential historical and biological context.

## mTOR complexes: mTORC1 and mTORC2

mTOR is an atypical serine/threonine protein kinase that serves as a key regulatory node linking growth factor signaling, nutrient availability, energy metabolism, and cellular stress responses (Saxton and Sabatini, 2017). Aberrant activation of the mTOR pathway is not simply reflected by increased phosphorylation of a single molecular target. Rather, it involves coordinated alterations in protein translation, anabolic metabolism, and cell survival programs mediated by two structurally and functionally distinct complexes, mTORC1 and mTORC2 (Yang et al., 2013).

mTORC1 is composed of mTOR, regulatory-associated protein of mTOR (Raptor), mammalian lethal with SEC13 protein 8 (mLST8), and regulatory components such as PRAS40 and DEPTOR. mTORC1 is relatively sensitive to rapamycin. It primarily regulates protein translation, ribosome biogenesis, lipid synthesis, nucleotide synthesis, and other anabolic processes. By contrast, mTORC2 consists mainly of mTOR, rapamycin-insensitive companion of mTOR (Rictor), mLST8, mammalian stress-activated protein kinase-interacting protein 1 (mSIN1), Protor1/2, and DEPTOR. Unlike mTORC1, mTORC2 is relatively insensitive to acute rapamycin treatment and is mainly involved in the phosphorylation of kinases such as AKT, SGK, and PKC (Yong et al., 2025). Through these downstream effectors, mTORC2 contributes to the maintenance of cell survival, anti-apoptotic signaling, cytoskeletal organization, and adaptive responses to cellular stress (Yang et al., 2013).

In CCA cells, upstream PI3K/AKT signaling, inflammatory stimulation, and metabolic stress activate mTOR. This activation does not merely induce changes in individual signaling molecules. Instead, it drives tumor cells into a sustained state of growth and metabolic adaptation (Ilyas et al., 2014). Among these downstream effects, mTORC1 promotes protein translation primarily through the p70S6K and 4E-BP1/eIF4E axes (Betz and Hall, 2013). p70S6K promotes the translation of ribosome-associated proteins and supports cell growth. Upon phosphorylation, 4E-BP1 dissociates from eIF4E, thereby relieving its inhibitory effect on cap-dependent translation initiation. Consequently, this process increases the synthesis of proteins involved in angiogenesis, cell-cycle progression, proliferation, and survival, such as VEGF, c-MYC, and Cyclin D1 (Okumura et al., 2020).

**FIGURE 1:**
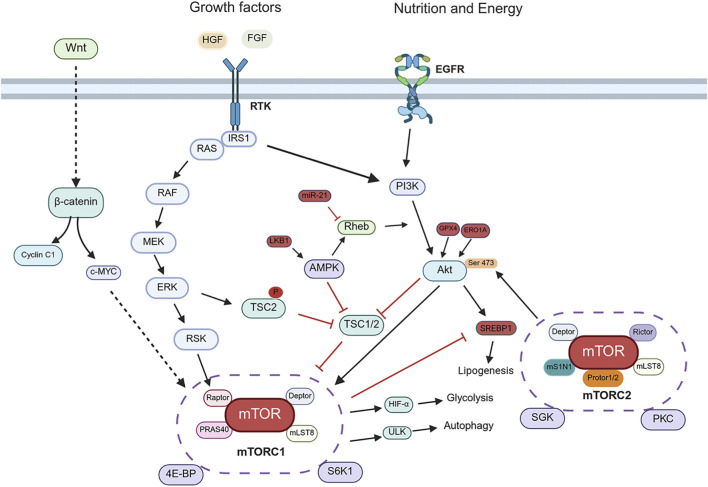
Overview of mTOR signaling and its upstream regulation in cholangiocarcinoma. This figure shows the growth factors, nutritional status, and cellular energy availability regulate mTOR signaling through RTK/IRS1/RAS/RAF/MEK/ERK, PI3K/Akt, AMPK, and Wnt/β-catenin signaling axes. mTOR functions mainly through two distinct complexes, mTORC1 and mTORC2. mTORC1, composed of mTOR, Raptor, mLST8, PRAS40, and Deptor, regulates protein synthesis, glycolysis, lipogenesis, and autophagy. mTORC2, containing mTOR, Rictor, mSIN1, mLST8, Protor1/2, and Deptor, modulates downstream effectors including Akt, SGK, and PKC. Arrows indicate activation, whereas red blunt-ended lines indicate inhibition. Created with BioRender.com.

As the proliferative demand of tumor cells increases, mTORC1 further regulates lipid, glucose, and nucleotide metabolism. This regulation provides the biosynthetic substrates required for cellular expansion. For example, mTORC1 promotes fatty acid and cholesterol synthesis through SREBP1/2. It also enhances glucose utilization by increasing the expression of glycolysis-related enzymes, and supports DNA replication and ribosome biogenesis by regulating purine and pyrimidine biosynthesis (Hagiwara et al., 2012; Peterson et al., 2011). Within this sustained growth state, mTORC2 enhances AKT activity through phosphorylation at Ser473 and regulates members of the AGC kinase family, including SGK and PKC. These events contribute to tumor cell survival, anti-apoptotic signaling, and adaptation to cellular stress. Importantly, mTORC2-mediated AKT activation can further influence mTORC1 activity through the AKT-TSC-Rheb axis. This cross-talk links survival signaling with anabolic metabolism (Hagiwara et al., 2012). Therefore, mTORC1 and mTORC2 should not be regarded as two isolated branches of the pathway. Rather, they cooperate to establish a signaling network that supports sustained proliferation, metabolic adaptation, and therapeutic tolerance in CCA cells (Figure 1).

## Upstream regulation of mTOR signaling

### PI3K/AKT/mTOR pathway

In CCA, growth factors such as hepatocyte growth factor (HGF) and fibroblast growth factor (FGF) bind to their corresponding receptor tyrosine kinases. This binding activating PI3K signaling and promotes AKT phosphorylation. Activated AKT subsequently transmits pro-growth signals to mTOR, contributing to tumor cell proliferation, survival, invasion, and angiogenesis (Voss et al., 2013; Ntanasis-Stathopoulos et al., 2020). In addition to enhanced receptor-mediated signaling, genetic alterations in components of the PI3K pathway may also contribute to aberrant mTOR activation. Previous studies have identified PIK3CA mutations in CCA samples, and exome sequencing analyses of a subset of intrahepatic cholangiocarcinoma (iCCA) cases have revealed somatic mutations in PI3K pathway-related genes, including PIK3CA, PIK3C2A, PIK3C2G, and PTEN (Ruzzenente et al., 2016). As a key negative regulator of the PI3K/AKT axis, loss of PTEN function can lead to sustained activation of this pathway (Cancer Genome Atlas Research Network et al., 2017). Beyond genetic alterations in PTEN itself, post-transcriptional regulation mediated by non-coding RNAs also contributes to the activation of AKT/mTOR signaling in CCA. For example, miR-21 has been shown to enhance AKT/mTOR signaling by suppressing PTEN expression (Ilyas et al., 2014). These findings suggest that upstream regulation of mTOR in CCA is highly complex. It involves not only genetic mutations but also epigenetic and post-transcriptional regulatory mechanisms.

### MAPK/ERK pathway

In addition to the canonical PI3K/AKT axis, the RAS/MAPK pathway represents another important branch of the upstream regulatory network of mTOR signaling (Carracedo et al., 2008; Nakamura et al., 2015). This pathway is typically initiated by receptor tyrosine kinase activation. It sequentially transduces signals sequentially through RAS, RAF, MEK, and ERK, thereby promoting cancer cell proliferation, migration, and metastasis. Previous studies have shown that genetic alterations in KRAS, NRAS, and BRAF can lead to aberrant activation of the MAPK pathway, with distinct mutational patterns across different anatomical subtypes of CCA (Ntanasis-Stathopoulos et al., 2020).

The RAS-RAF-MEK-ERK cascade primarily influences mTOR signaling by indirectly regulating mTORC1 (Lu et al., 2021). Earlier studies have demonstrated that RAS can bind to and activate PI3K (Gupta et al., 2007), suggesting potential crosstalk between the RAS/MAPK and PI3K/AKT pathways. Additionally, ERK can phosphorylate TSC2. This phosphorylation weakens the inhibitory effect of the TSC1/2 complex on Rheb, which promotes Rheb-mediated activation of mTORC1 (Miyazaki and Takemasa, 2017). ERK and its downstream effector RSK can also act on Raptor, a core component of mTORC1, thereby enhancing mTORC1 signaling output toward downstream substrates (Carrière et al., 2008). These findings indicate that the RAS/MAPK pathway is not completely independent of mTOR signaling, but can converge on the mTORC1 regulatory network through key nodes such as TSC2, Rheb, and Raptor.

Notably, the interaction between mTORC1 and the RAS/MAPK pathway is not unidirectional, but involves complex feedback regulation. Following mTORC1 activation, its downstream effector S6 kinase 1 (S6K1, also known as p70S6K) can exert negative feedback on RTK/IRS-1/PI3K signaling pathway. This feedback loop limits sustained upstream growth factor signaling (Carracedo et al., 2008). In contrast, mutations that directly activate mTOR signaling, such as alterations in TSC1/2 and CTNNB1, appear to be relatively uncommon in iCCA. Whether frequent molecular alterations in TP53, IDH1/2, and SMAD4 can directly activate mTOR signaling remains to be further clarified (Lu et al., 2021). These observations suggest that mTOR activation in CCA is more likely driven by upstream signaling convergence and network-level dysregulation rather than direct genetic alterations in core mTOR components.

### Other upstream regulators

Nutrient availability and cellular energy status are also important regulators of mTORC1 activity. mTORC1 can be modulated by amino acids, glucose, and cellular energy levels, among which amino acids such as leucine, glutamine, and arginine play critical roles in mTORC1 activation (Jewell et al., 2013). Under conditions of energy deprivation or nutrient scarcity, the LKB1/AMPK axis suppresses mTORC1 activity by inhibiting Raptor or activating the TSC complex. This process limits protein translation and anabolic metabolism and contributes to the induction of autophagy (Nicklin et al., 2009).

Studies in liver cancer models have further suggested that c-MYC-mediated enhancement of amino acid transport and Wnt/β-catenin-glutamine synthetase (GS)-mediated glutamine metabolism may intersect with mTORC1 activity (Nicklin et al., 2009). Although these metabolic regulatory mechanisms have been more extensively investigated in hepatocellular carcinoma and liver-based models, direct evidence in CCA remains relatively limited. Nevertheless, they may provide a useful framework for understanding how CCA cells maintain metabolic adaptation and sustained survival under hypoxic, inflammatory, and nutrient-stressed conditions. These observations further highlight the context-dependent nature of mTORC1 regulation and underscore the importance of metabolic inputs in shaping its activity in CCA.

## Downstream biological effects of mTOR signaling

Tumorigenesis is highly dependent on cellular metabolic reprogramming, which can arise as a direct consequence of oncogenic alterations and as an adaptive response to malignant progression. By reshaping energy acquisition and biosynthetic utilization, tumor cells are able to survive and proliferate under hostile microenvironmental conditions (Pavlova and Thompson, 2016; Paz et al., 2017). In CCA, metabolic reprogramming involves multiple interconnected processes, including glycolysis, lipid metabolism and amino acid metabolism (Raggi et al., 2022).

As a central nutrient- and growth factor-sensing kinase, mTOR plays a pivotal role in coordinating metabolic homeostasis in cancer cells. In particular, mTORC1 integrates signals from growth factors, nutrient availability, cellular energy status, and the tumor microenvironment to regulate protein synthesis, lipid biosynthesis, glucose metabolism, and cell proliferation (Hagiwara et al., 2012; Pant et al., 2020). Thus, mTOR-driven metabolic reprogramming represents a key mechanism by which CCA cells sustain growth, survival, and adaptation to metabolic stress.

### Glucose metabolism

Tumor cells often exhibit increased glucose uptake and enhanced glycolytic flux, a metabolic phenotype that provides the biological basis for ^18^F-FDG PET imaging (Ikeno et al., 2018). Although many tumors retain mitochondrial oxidative phosphorylation as an important source of ATP production, enhanced aerobic glycolysis enables cancer cells to generate glycolytic intermediates required for nucleotide, amino acid, and lipid biosynthesis, thereby supporting rapid proliferation and adaptation to hostile microenvironments (Zheng, 2012). In CCA, glucose metabolic reprogramming is characterized by increased glucose transport, accelerated glycolytic flux, and lactate accumulation, involving key molecules such as GLUT1, HK2, PKM2, and LDHA (Kubo et al., 2014; Thamrongwaranggoon et al., 2017; Qian et al., 2020; Hoxhaj and Manning, 2020).

mTOR signaling is a central regulator of this glycolytic phenotype. Activation of the PI3K/AKT/mTOR axis promotes glucose uptake and glycolytic metabolism through both transcriptional and post-translational mechanisms (Hoxhaj and Manning, 2020). On one hand, mTORC1 enhances the expression of nutrient transporters, particularly GLUT1, which increases glucose availability for tumor cells. On the other hand, mTORC1 can promote the activity of transcription factors such as HIF-1α and c-Myc, which transcriptionally regulate glycolysis-related genes, including GLUT1, HK2, PKM2, LDHA, phosphofructokinase (PFK), and enolase 1 (ENO1) (Pant et al., 2020; Wu et al., 2015). Through these mechanisms, mTOR signaling links growth factor stimulation and nutrient sensing to enhanced glycolytic flux and lactate production.

In CCA, this regulatory axis has been linked to metabolic adaptation, therapeutic resistance, and glycolysis-associated malignant progression. For example, CCA cells can evade sorafenib-mediated inhibition of the RAF/MEK/ERK by activating the AKT/mTOR pathway. This suggests that mTOR signaling actively drives metabolic adaptation and therapeutic resistance (Conciatori et al., 2018). Furthermore, Hori et al. reported that glutathione peroxidase 4 (GPX4) may promote glucose metabolic reprogramming in CCA by activating the AKT/mTOR axis. This activation is reflected by increased expression of GLUT1, HIF-1α, and LDHA and enhanced ^18^F-FDG uptake. Clinically, GPX4-mediated metabolic dysregulation was associated with larger tumor size, lymph node metastasis, and poor prognosis (Hori et al., 2024). In addition, Zhu et al. found that the long non-coding RNA NKILA enhances the glycolytic phenotype of iCCA cells by regulating PKM2 expression and nuclear localization, which in turn activates mTOR signaling. This NKILA-PKM2-mTOR axis not only suppresses autophagy and maintains stable PD-L1 expression but also reduces CD8^+^ T cell cytotoxicity. These findings suggest that mTOR-related glycolytic reprogramming in CCA is not only involved in metabolic adaptation but may also cooperate with immune evasion and treatment resistance (Zhu et al., 2025). Collectively, these observations highlight the central role of mTOR signaling in integrating metabolic reprogramming with therapeutic resistance and immune modulation in CCA.

### Lipid metabolism

Lipid metabolic reprogramming is another important component of metabolic adaptation in CCA. Tumor cells can enhance *de novo* fatty acid synthesis or increase exogenous lipid uptake to provide substrates for membrane biogenesis, energy storage, and lipid-mediated signaling (Snaebjornsson et al., 2020). *De novo* fatty acid synthesis mainly depends on key enzymes such as acetyl-CoA carboxylase (ACC) and fatty acid synthase (FASN), and is regulated by sterol regulatory element-binding proteins 1/2 (SREBP1/2) and oncogenic signaling pathways, including the PI3K/AKT/mTOR axis. In this context, mTOR does not regulate lipid metabolism in a single linear manner. Rather, mTORC1 and mTORC2 may coordinate lipid metabolic processes through distinct but interconnected mechanisms.

However, lipid metabolic patterns in CCA appear to be heterogeneous. Some studies have shown that iCCA cells are not highly sensitive to FASN inhibition, suggesting that they may compensate for reduced *de novo* fatty acid synthesis by increasing exogenous lipid utilization through fatty acid transport-related molecules such as FATP1 (Li et al., 2016). Additionally, fatty acid-binding protein 5 (FABP5) is highly expressed in eCCA and correlates with poor prognosis. Conversely, FABP4 may mediate adipocyte-induced invasion, migration, and epithelial–mesenchymal transition (EMT) in CCA cells (Nie et al., 2017). These findings indicate that CCA lipid metabolism involves not only intrinsic fatty acid synthesis but also lipid uptake and lipid trafficking within the tumor microenvironment.

The AKT/mTOR pathway is closely linked to SREBP1-mediated lipogenesis. Inhibition of this pathway is accompanied by reduced expression of SREBP1 and its downstream lipogenic enzymes, including ACC and FASN. Zhou et al. reported that cordycepin suppresses AKT/mTOR signaling activity in CCA cells and downregulates the SREBP1-mediated lipogenic program, as reflected by decreased ACC1 and FASN expression and reduced levels of triglycerides, total cholesterol, very-low-density lipoprotein (VLDL), and non-esterified fatty acids (NEFAs). Further evidence suggests that endoplasmic reticulum oxidoreductase 1 alpha (ERO1A) may act upstream of the PI3K/AKT/mTOR pathway and promote fatty acid synthesis and lipid accumulation through activation of the AKT/mTOR/SREBP1 axis (Zhou et al., 2023). ERO1A is highly expressed in iCCA and can activate AKT in a reactive oxygen species (ROS)-dependent manner, thereby enhancing mTORC1 activity and forming a positive feedback regulatory loop (Wang et al., 2024).

In addition to ERO1A, MAL2 has been shown to promote lipid accumulation in iCCA through the EGFR/PI3K/AKT/SREBP1 axis. Mechanistically, MAL2 maintains EGFR membrane localization and inhibits its endocytic degradation. This enhances PI3K/AKT/SREBP1 signaling and promotes the expression of fatty acid synthesis-related genes, such as FASN and stearoyl-CoA desaturase (SCD) (Huang et al., 2024). Non-coding RNAs also participate in mTOR-related lipid metabolic regulation in CCA. Ma et al. found that lncRNA HAGLROS is highly expressed in iCCA and associated with poor prognosis. Knockdown of HAGLROS suppresses CCA cell proliferation, migration, and invasion, at least in part by inhibiting mTOR-related lipid metabolic programs (Ma et al., 2020). In addition, miR-199a-3p can directly target mTOR and suppress its signaling activity (Li et al., 2017). Overall, lipid metabolic reprogramming in CCA involves both SREBP1/ACC/FASN-mediated *de novo* fatty acid synthesis and FATP/FABP-mediated exogenous lipid uptake. AKT/mTOR signaling contributes to this process mainly by regulating SREBP1 and its downstream lipogenic program and coordinating lipid metabolic flexibility in response to microenvironmental cues.

### Amino acid metabolism

Amino acid metabolic reprogramming is another important component of metabolic adaptation in CCA. Tumor cells reshape amino acid metabolism not only to provide biosynthetic substrates for malignant proliferation but also to regulate mTOR signaling, thereby supporting cell growth and metabolic adaptation. Amino acids such as glutamine and leucine are important activators of mTORC1. In turn, mTORC1 downstream effectors, including ATF4 and c-Myc, can upregulate the expression of amino acid transporters and key metabolic enzymes, thereby enhancing amino acid uptake and metabolic capacity in tumor cells. This establishes a positive regulatory relationship between amino acid metabolism and mTOR signaling (Torrence et al., 2021; Hua et al., 2026). Although mTORC2 is not considered the major complex directly responsible for amino acid sensing, it regulates cell proliferation, survival, and therapeutic resistance through downstream effectors such as AKT, SGK, and protein kinase C, thereby cooperating with mTORC1 in tumor metabolic adaptation (Hagiwara et al., 2012).

Specifically, amino acid transporters such as L-type amino acid transporter 1 (LAT1) are upregulated in CCA, facilitating amino acid uptake and promoting tumor cell invasion and migration. Glutamine metabolism represents a key link between amino acid metabolic reprogramming and mTOR activation (Chen and Cui, 2015). Studies have shown that cancer-associated fibroblasts in the tumor microenvironment can maintain mTORC1 activity in CCA cells by secreting glutamine, whereas inhibition of glutamine transport or glutaminase activity reduces mTOR signaling. In this context, mTOR-related pathways may also regulate the expression of key enzymes involved in serine–glycine one-carbon metabolism, such as PHGDH and PSAT1, thereby further supporting the biosynthetic demands of CCA cells (Zhu et al., 2024). Glutamine dependence is a prominent metabolic feature of CCA. Through glutaminolysis, glutamine can be converted into α-ketoglutarate, replenishing tricarboxylic acid cycle intermediates and supporting both enhanced glycolysis and *de novo* lipid synthesis (Metallo et al., 2011). Wappler et al. reported that long-term glutamine deprivation reduces chemoresistance in extrahepatic CCA cells, possibly through suppression of c-Myc expression (Wappler et al., 2020). These findings suggest that glutamine metabolism contributes not only to bioenergetic and biosynthetic adaptation but also to therapeutic resistance in CCA.

Amino acid metabolic reprogramming can also influence the AKT/mTOR pathway through redox homeostasis. Thanee et al. found that CD44 maintains the availability of substrates required for glutathione synthesis, including cysteine, glutamate, and glutamine, thereby enhancing the antioxidant capacity of CCA cells. Knockdown of CD44 reduced AKT and mTOR phosphorylation and impaired CCA cell proliferation (Thanee et al., 2021). These findings indicate that amino acid metabolism may regulate mTOR activity not only through nutrient-sensing mechanisms but also through redox-dependent signaling pathways. More recently, the SIRT6-GLUL axis has been identified as an additional mechanism involved in glutamine metabolic reprogramming in intrahepatic CCA. SIRT6 promotes GLUL transcription and inhibits ubiquitination-mediated degradation of GLUL protein, thereby sustaining intracellular glutamine synthesis (Zhang M. et al., 2025). This pathway may function as an important downstream branch of mTOR-associated glutamine metabolic regulation, further amplifying the metabolic advantages of tumor cells. However, the specific regulatory relationship between mTOR signaling and the SIRT6–GLUL axis in intrahepatic CCA requires further investigation.

## mTOR-mediated malignant phenotypes

### Proliferation

The AKT/mTOR signaling axis is frequently activated in CCA and represents an important mechanism driving malignant proliferation. Chung et al. performed multiplex tissue immunoblotting in 221 extrahepatic CCA specimens and found that the expression levels of p-AKT and p-mTOR were significantly higher in tumor tissues than in normal bile duct epithelium and dysplastic bile duct epithelium. Moreover, p-AKT expression was significantly correlated with p-mTOR expression, supporting the activation of the AKT/mTOR axis in eCCA (Chung et al., 2009). As a central hub regulating cell growth and proliferation, mTOR integrates diverse upstream signals, including membrane-associated proteins, transcriptional regulators, and ubiquitination-related modifiers. Through the mTORC1/p70S6K/RPS6 axis and the mTORC2/AKT axis, mTOR signaling regulates protein synthesis, cell-cycle progression, and metabolic adaptation, thereby promoting sustained proliferation of CCA cells.

Mechanistically, a bidirectional regulatory relationship has been identified between the ion channel protein TMEM16A and mTOR. TMEM16A and mTOR cooperate to maintain the proliferative activity of CCA cells by regulating mTORC1-mediated RPS6 phosphorylation and mTORC2-dependent AKT activation, ultimately contributing to malignant progression (Kulkarni et al., 2023). In addition to membrane-associated regulation, mTOR signaling is also influenced by intracellular transcriptional programs. The Merlin/YAP/c-Myc signaling axis can sustain mTOR activation under high-density culture conditions. This allows CCA cells to escape contact inhibition, bypass G0/G1 arrest, and acquire unrestricted proliferative capacity. These findings suggest that aberrant transcriptional regulation can promote CCA cell proliferation through mTOR-dependent mechanisms (Luo et al., 2017). Furthermore, the ubiquitination-related protein TRIM59 positively regulates phosphorylation-mediated activation of the PI3K/AKT/mTOR pathway and promotes tumor cell proliferation by accelerating cell-cycle progression (Shen et al., 2019). Li et al. further revealed a link between mTOR signaling and cell-cycle-dependent proliferation in intrahepatic CCA. Mechanistically, HMGA1 promotes CCND1 expression and activates the PI3K/AKT/mTOR pathway. This enhances enhancing iCCA cell proliferation and colony-forming ability. Both the CDK4/6 inhibitor palbociclib and the PI3K/mTOR inhibitor PF-04691502 suppress iCCA cell proliferation. Compared with either monotherapy, combined treatment more effectively downregulates PI3K/mTOR pathway activity, blocks the G1/S phase transition, and strengthens the anti-proliferative effect (Li et al., 2023).

### EMT, invasion, and metastasis

Tumor metastasis is a complex and multistep biological process. Epithelial–mesenchymal transition (EMT) is a highly conserved cellular program that endows epithelial cells with migratory and invasive properties. This process is characterized by changes in epithelial and mesenchymal markers and is closely associated with malignant tumor progression (Pastushenko and Blanpain, 2019; Manfioletti and Fedele, 2023). Clinical histological studies have shown that aberrant activation of the mTOR pathway is common in CCA tissues. Wang et al. performed immunohistochemical analysis of 77 intrahepatic CCA samples and found that p-mTOR, p-4E-BP1, and p-p70S6K were highly expressed in tumor tissues. Notably, high p-4E-BP1 expression was closely associated with poor prognosis, indirectly suggesting that mTOR pathway activation may contribute to malignant progression in CCA (Wang et al., 2012). In addition, KIN17 has been reported to markedly increase mTOR phosphorylation in CCA, mainly regulating cell migration and invasion rather than directly promoting proliferation (Yang et al., 2026).

Both mTORC1 and mTORC2 play important roles in cancer cell migration and invasion (Gulhati et al., 2011; Guenzle et al., 2020). Mechanistically, mTORC1 can regulate cytoskeletal organization and motility through downstream effectors such as S6K1 and 4E-BP1, whereas mTORC2 is more closely involved in cytoskeletal remodeling through phosphorylation of proteins such as filamin A. In addition, mTOR signaling may modulate matrix metalloproteinase (MMP) activity, thereby influencing extracellular matrix degradation, migration, and invasive capacity. Inhibition of mTORC1/2 can also interfere with ERK and p38 activation, thereby disrupting MAPK pathway signaling. Consistent with these mechanisms, dual targeting of mTORC1 and mTORC2 with the kinase inhibitor OSI-027 significantly reduces migration and invasion of iCCA cells without markedly affecting cell viability or proliferation (Joechle et al., 2022). OSI-027 potently inhibits both mTORC1 and mTORC2 and broadly suppresses tumor cell motility by modulating RhoA, Rac1, and cytoskeletal remodeling. In contrast, the classical mTOR inhibitor rapamycin exhibits time-dependent effects on mTOR complex regulation. Short-term rapamycin treatment mainly inhibits mTORC1 and may induce feedback activation of the PI3K/AKT pathway, whereas prolonged exposure can disrupt mTORC2 complex assembly.

In sarcomatoid CCA, the mTOR/AKT pathway is aberrantly activated. Rapamycin suppresses EMT and peritoneal dissemination by blocking mTORC2-mediated phosphorylation of STAT3 at Ser727. This blockade downregulates both the EMT-related transcription factor Twist1 and the matrix metalloproteinase MMP2 at the mRNA and protein levels (Hong et al., 2013). In addition, Fyn has been shown to be overexpressed in CCA cell lines. Knockdown of Fyn induces AMPK phosphorylation and modulates the AMPK/mTOR signaling pathway. The AMPK inhibitor Compound C can reverse the inhibitory effects of Fyn knockdown on CCA cell migration and invasion, suggesting that targeting Fyn activity may represent a potential therapeutic strategy for CCA (Lyu et al., 2018).

In recent years, non-coding RNAs, especially circular RNAs, have been shown to regulate mTOR signaling through competing endogenous RNA mechanisms. In intrahepatic CCA, circ 0084927 acts as a sponge for miR-4725-5p, upregulates PDPK1 expression, and further activates the AKT/mTOR pathway, thereby enhancing tumor cell proliferation and metastatic potential (Xie et al., 2025). Zhang et al. identified CARD9 as significantly upregulated in CCA tissues based on GEO and TCGA database analyses. Experimental validation showed that CARD9 binds to THEM4, a negative regulator of AKT. This binding relieves THEM4-mediated inhibition of AKT/mTOR signaling. In parallel, CAED9 activates the NF-κB/NLRP3 inflammatory pathway. These combined actions promote the release of inflammatory factors like IL-17A and activate Hedgehog signaling, thereby contributing to CCA progression (Zhang et al., 2024).

Doublecortin-like kinase 1 (DCLK1), a member of the protein kinase superfamily and doublecortin family, is also significantly upregulated in CCA tissues. High DCLK1 expression suppresses E-cadherin and upregulates mesenchymal markers, including vimentin, N-cadherin, and Snail. Moreover, DCLK1 activates the PI3K/AKT/mTOR signaling pathway, thereby inducing EMT and promoting malignant progression of CCA cells (Liang et al., 2024). Collectively, these findings indicate that diverse regulatory molecules, including non-coding RNAs, inflammatory adaptors, membrane-associated kinases, and EMT-related signaling proteins, can converge on the mTOR pathway to promote EMT, invasion, and metastasis in CCA.

### Autophagy

Autophagy is a conserved physiological process by which cytoplasmic contents are delivered to lysosomes for degradation, playing an essential role in maintaining cellular energy homeostasis and resistance to nutrient stress (Mizushima, 2007). The role of autophagy in cancer is context-dependent. Under conditions of nutrient deprivation or mitochondrial damage, autophagy can provide metabolic substrates for tumor cells, thereby supporting tumor growth and exerting pro-tumorigenic effects (Chung et al., 2020). Conversely, during early tumorigenesis, autophagy may prevent malignant transformation by removing damaged organelles, alleviating oxidative stress, suppressing inflammation, and maintaining genomic stability (Liu et al., 2023).

As an evolutionarily conserved protein kinase, mTOR senses nutrient availability and cellular stress signals and coordinates the dynamic balance between cell growth and autophagic activity. In CCA, multiple regulatory mechanisms, including the PI3K/AKT/mTOR and AMPK signaling pathways, participate in autophagy regulation. Atractylodin (ATD), a natural bioactive compound, has been shown to promote autophagy in CCA cells by inhibiting PI3K/AKT/mTOR signaling and upregulating autophagy-related proteins such as LC3-II (Acharya et al., 2021). This observation is consistent with the findings of Pongking et al., who reported that cannabidiol (CBD) can induce autophagy in CCA cells (Pongking et al., 2024).

However, autophagy regulation in CCA is not limited to the classical AKT/mTOR-dependent model. Compound C, an AMPK inhibitor, induces autophagy in CCA cells through a mechanism that appears to be independent of the conventional AMPK–mTOR axis. Compound C increases AKT and p70S6K phosphorylation while reducing mTOR-mediated phosphorylation of ULK1 at Ser757. It also activates the p38 MAPK pathway to induce autophagy (Zhao et al., 2018). These findings suggest that CCA cells harbor multiple parallel and partially independent regulatory networks controlling autophagy.

Importantly, autophagy is a continuous and dynamic degradation process. Autophagosome accumulation may result not only from enhanced autophagy initiation but also from impaired downstream degradation or blockade of autophagic flux. Previous studies have shown that mTOR inhibition does not necessarily induce complete autophagic degradation; in some contexts, it may impair autophagic flux and lead to autophagy blockade (Cinà et al., 2012). Meanwhile, AMPK activation can promote autophagosome formation, further aggravating autophagosome accumulation.

Albendazole (ABZ) has been reported to regulate AMPK/mTOR signaling and inhibit p70S6K activity. While ABZ induces autophagy initiation and promotes autophagosome formation, it also causes simultaneous accumulation of LC3B-II and p62, indicating blockade of the autophagic degradation process (He Q. et al., 2022). These findings highlight the importance of distinguishing autophagy activation from effective autophagic flux when evaluating mTOR-related autophagy regulation in CCA.

Current evidence suggests that autophagy-targeted intervention has potential antitumor effects, but its biological consequences are highly context-dependent (Yang et al., 2025). In recent years, endoplasmic reticulum stress (ER stress) has been shown to induce autophagy through activation of the unfolded protein response (UPR). Mechanistically, ER stress can also participate in autophagy regulation through the mTOR/S6K1 signaling pathway. Under persistent ER stress, autophagy may shift from an adaptive survival response to autophagy-dependent cell death (Galluzzi et al., 2018). Nevertheless, the role of autophagy in cell death regulation remains controversial. Increasing evidence suggests that under conditions such as nutrient deprivation, anticancer drug treatment, and ER stress, high levels of autophagic flux may induce multiple forms of programmed cell death, including apoptosis and ferroptosis (Zhou et al., 2020; Gao et al., 2016).

### Apoptosis

Apoptosis is a highly conserved form of programmed cell death that plays an essential physiological role in eliminating abnormal cells and maintaining tissue homeostasis (Singh and Lim, 2022). Aberrant activation of mTOR signaling can regulate cell growth, metabolic reprogramming, and survival signaling, thereby enhancing the ability of tumor cells to adapt to hostile microenvironmental conditions and evade apoptosis. In CCA, several studies have shown that suppression of the AKT/mTOR axis can restore apoptotic sensitivity. Sawasdee et al. demonstrated that an ethanol extract of the microalga Chlorella inhibited AKT/mTOR signaling in CCA cells. This effect was accompanied by downregulation of pro-caspase-3, pro-caspase-8, pro-caspase-9, and Bcl-2, activation of the caspase cascade, and induction of apoptosis (Sawasdee et al., 2023).

Notably, AKT/mTOR signaling does not function as an isolated survival pathway, but interacts extensively with MAPK, STAT3, AMPK, and endoplasmic reticulum stress-related pathways. Jia et al. reported that baicalin induced caspase-dependent apoptosis in QBC939 cells in a time- and dose-dependent manner. Mechanistically, baicalin activated AMPK and promoted raptor phosphorylation, thereby blocking mTORC1 downstream p70S6K signaling. This was associated with increased expression of cleaved caspase-3 and cleaved PARP, ultimately contributing to its antitumor effect (Jia et al., 2022). The complex network structure surrounding mTOR signaling may explain why single-target interventions often fail to produce durable antitumor responses. Although rapamycin can inhibit mTOR signaling, rapamycin monotherapy may trigger compensatory AKT phosphorylation, thereby weakening its antitumor efficacy. Combined treatment with rapamycin and salubrinal partially reverses this effect and synergistically suppresses tumor cell proliferation. In addition, rapamycin can increase the expression of the anti-apoptotic protein Bcl-xL *in vivo*, whereas salubrinal effectively counteracts this undesirable change. Through dual regulation of AKT activation and Bcl-xL expression, salubrinal may optimize the pro-apoptotic effect of mTOR inhibition (Zhao et al., 2016).

Furthermore, apoptosis regulation in the *in vivo* tumor microenvironment is influenced by multiple stress conditions, including hypoxia, nutrient deprivation, oxidative stress, and endoplasmic reticulum stress. These factors may reshape apoptotic signaling and interfere with the therapeutic efficacy of targeted agents (Sun et al., 2026; Wandee et al., 2021; Chen et al., 2024). Therefore, mTOR-related apoptosis regulation in CCA should be understood not as a linear pathway, but as a stress-responsive survival network involving feedback activation, anti-apoptotic proteins, and crosstalk with other signaling axes.

## Therapeutic targeting of mTOR signaling

### mTOR inhibitors: monotherapy and combination strategies

The development of mTOR inhibitors originated from the natural product rapamycin, which was initially isolated from the actinomycete *Streptomyces* hygroscopicus and first recognized for its antifungal activity. With increasing understanding of its molecular mechanism, mTOR inhibition was later found to exert antitumor activity in multiple cancer types. Rapamycin binds to FK506-binding protein 12 (FKBP12) to form a complex that allosterically inhibits mTOR complex 1 (mTORC1), thereby suppressing mTORC1-dependent signaling. Because rapamycin and its analogs mainly inhibit mTORC1 and do not directly suppress mTORC2, they are generally classified as first-generation mTOR inhibitors.

First-generation mTOR inhibitors mainly include sirolimus and its derivatives, such as everolimus (RAD001). However, selective inhibition of mTORC1 without direct or sustained inhibition of mTORC2 represents a major therapeutic limitation. It frequently triggers compensatory activation of the PI3K–AKT pathway through the S6K1–IRS1 feedback loop. This feedback ultimately weakens the overall antitumor efficacy of these agents (Moolthiya et al., 2014; Wu et al., 2019; Zhang H. et al., 2025). To overcome these limitations, second-generation mTOR inhibitors, also known as pan-mTORC1/2 inhibitors or TORKIs, have been developed. These ATP-competitive inhibitors directly target the kinase domain of mTOR and suppress both mTORC1 and mTORC2 signaling. For example, MLN0128 has shown stronger antitumor activity than first-generation inhibitors in preclinical studies (Slotkin et al., 2015). Several compounds, including AZD-2014, MLN0128, and OSI-027, have been investigated in clinical or preclinical studies to evaluate their therapeutic relevance in cancer treatment (Joechle et al., 2022; Liu et al., 2012; Zhang et al., 2017). Although dual mTOR inhibitors have shown therapeutic potential in multiple cancer models (Li et al., 2021; Mortazavi et al., 2022), durable tumor control with monotherapy remains difficult, largely because of feedback activation and compensatory signaling pathway reprogramming. Loss of ARID1A expression has been reported to increase the sensitivity of cancer cells to PI3K and AKT inhibitors (Samartzis et al., 2014). Consistently, ARID1A-deficient cholangiocarcinoma cells showed increased sensitivity to the AKT inhibitor MK-2206 (Tessiri et al., 2022).

In this context, combined blockade of tumor proliferation-related pathways has become an important strategy to improve therapeutic efficacy. MLN0128, as a second-generation pan-mTOR inhibitor, can simultaneously inhibit mTORC1 and mTORC2 and induce apoptosis in AKT/Yap-driven intrahepatic CCA models. However, its inhibitory effect on tumor cell proliferation appears limited, producing only transient disease stabilization and temporary decreases in the tumor marker CA19-9 without markedly delaying tumor progression (Rizell et al., 2008). Further studies have combined the CDK4/6 inhibitor palbociclib with MLN0128 to suppress AKT/YapS127A-dependent proliferation. This combination showed stronger growth-inhibitory effects in both *in vitro* and *in vivo* ICC models (Song et al., 2019). In recent years, combination strategies targeting mTOR signaling have attracted increasing attention in CCA, and representative therapeutic combinations and their mechanisms are summarized in Table 1.

**TABLE 1:** Representative combination strategies targeting mTOR signaling in cholangiocarcinoma.

Combination	Pathways	Mechanisms	Main effects	Ref.
MLN0128 + Palbociclib	pan-mTOR/CDK4/6–Rb cell-cycle axis	Combines MLN0128-induced mTOR blockade with Palbociclib-driven CDK4/6–Rb cell-cycle arrest; enhances mTOR downstream inhibition, further reduces Rb phosphorylation, and prevents Palbociclib-induced Cyclin D1 upregulation	Synergistically suppresses ICC proliferation *in vitro* and induces tumor regression in AKT/YapS127A ICC mice	Song et al. (2019)
RAD001 + MK-2206	mTORC1/pan-AKT	MK-2206 counteracts RAD001-induced AKT feedback activation, enabling vertical AKT–mTOR pathway blockade and synergistic inhibition of CCA cell proliferation, G0/G1 progression, and colony formation	Synergistically inhibits CCA proliferation *in vitro* and suppresses xenograft tumor growth *in vivo*	Ewald et al. (2013)
BEZ235 + JQ1	PI3K/AKT/mTOR + BET–YAP/c-Myc transcriptional axis	BEZ235 suppresses PI3K/AKT/mTOR signaling but induces resistance-associated YAP and c-Myc upregulation via reduced LATS1 phosphorylation and JQ1 inhibits BET-dependent YAP/c-Myc transcription, restoring sensitivity to PI3K/mTOR blockade	Suppresses ICC proliferation, invasion, and progression *in vitro* and in AKT/YAP-driven ICC mice	Miao et al. (2021)
AZD6244 + MK-2206	mTORC1/2–AKT	MK-2206 blocks residual AKT activity that rebounds through AKT T308 phosphorylation after AZD8055-mediated mTORC1/2 inhibition, enabling vertical AKT–mTOR pathway blockade	Strongly synergistic suppression of CCA proliferation and G1 cell-cycle progression *in vitro*	Ewald et al. (2014)
AZD6244 + MK-2206	MEK–ERK + AKT	AKT inhibition counteracts compensatory AKT-mediated survival signaling associated with MEK blockade and restores sensitivity in AZD6244-resistant CCA cells	Synergistically suppresses proliferation and reverses acquired MEK inhibitor resistance *in vitro*	Ewald et al. (2014)
AZD6244 + AZD8055	MEK–ERK + mTORC1/2	mTORC1/2 inhibition limits PI3K/AKT/mTOR pathway compensation after MEK inhibition, although synergy is weaker than AKT–mTOR vertical blockade in parental CCA cells	Suppresses proliferation and reverses acquired MEK inhibitor resistance *in vitro*	Ewald et al. (2014)
NVP-BEZ235 + NVP-AUY922	PI3K/mTOR + HSP90	NVP-AUY922 destabilizes HSP90 client signaling proteins including Akt and induces ER stress, while NVP-BEZ235 blocks PI3K/Akt/mTOR signaling; the combination prolongs pathway inhibition and amplifies ROS accumulation, GSH depletion, mitochondrial dysfunction, and ER stress–mediated apoptosis	Synergistically induces apoptosis in CCA cells and promotes tumor regression in a TAA-induced CCA rat model	Chen et al. (2014)
Sorafenib + Everolimus with mTORC2/Rictor suppression	RAF–MEK–ERK + mTORC1/mTORC2–AKT	Sorafenib induces an mTORC2-dependent AKT Ser473 escape signal that limits RAF–MEK–ERK blockade; Rictor/mTORC2 suppression blocks this survival pathway, while everolimus adds mTORC1 inhibition to strengthen combined AKT/mTOR and RAF–MEK–ERK targeting	Enhances growth inhibition and apoptosis in RBE CCA cells, with the strongest effect under mTORC2 disassembly	Yokoi et al. (2018)

### Natural small-molecule modulators of mTOR signaling

In recent years, although combination strategies targeting the mTOR pathway have shown certain antitumor effects in CCA, their clinical application remains limited by toxicity, therapeutic resistance, and cost-related concerns. Therefore, the development of novel therapeutic approaches with multi-target regulatory potential and favorable safety profiles has become an area of increasing interest. Among these approaches, natural product-derived small molecules have attracted attention because of their structural diversity and broad biological activities.

In CCA, the specific antitumor effects of natural small-molecule compounds are largely mediated through modulation of the PI3K/AKT/mTOR signaling pathway and its related molecular networks. Rather than acting solely as direct mTOR inhibitors, these compounds often regulate mTOR-associated signaling in a context-dependent manner. They may suppress mTOR pathway activity, induce autophagy, promote apoptosis, modulate tumor metabolism, and simultaneously affect MAPK, STAT3, ROS-related, or endoplasmic reticulum stress-associated pathways. Through these multi-layered mechanisms, natural small-molecule modulators may inhibit CCA initiation, progression, and therapeutic resistance. Despite these promising preclinical findings, translating these natural compounds into clinical practice requires systematically addressing several critical issues, including the lack of clinical validation, unclear pharmacokinetics, potential toxicity, and the necessity for standardized active components. Representative compounds and their mechanisms of action are summarized in Table 2.

**TABLE 2:** Effects of natural compounds in mTOR signaling pathway in cholangiocarcinoma.

Compounds	Souces	Models	Mechanisms	Ref.
2-dodecyl-6-methoxycyclohexa-2,5-diene-1,4-dione (DMDD)	Plant-derived quinone extracted from Populus tremula root	*In vitro* and *in vivo* (QBC939 cells; QBC939 xenograft mouse model)	Downregulates PI3K/AKT/mTOR signaling and induces Beclin-1/LC3-associated autophagy, leading to cell-cycle arrest and reduced proliferation, migration, and invasion	He Y. et al. (2022)
Cannabidiol (CBD)	Cannabis-derived phytocannabinoid from Cannabis sativa L.	*In vitro* and *in vivo* (KKU-055, KKU-100, KKU-213B cells; KKU-100 xenograft model)	Inhibits PI3K/AKT/mTOR signaling and induces LC3B/p62-associated autophagy, accompanied by mitochondrial ROS accumulation, G0/G1 arrest, and p53/p21-mediated senescence	Pongking et al. (2024)
Albendazole (ABZ)	Repurposed synthetic antiparasitic drug; benzimidazole derivative	*In vitro* and *in vivo* (RBE and FRH-0201 cells; HIBEPIC control cells; RBE xenograft mouse model)	Activates AMPK/mTOR signaling to initiate Beclin-1-independent autophagosome formation, while blocking autophagosome–lysosome fusion and autophagic flux, thereby promoting caspase-dependent apoptosis in CCA cells	He Q. et al. (2022)
Adenosine	Endogenous purine nucleoside	*In vitro* (HuCCA-1 and RMCCA-1 cells; MMNK-1 control cells)	Activates AMPK signaling and induces LC3-II-associated autophagy via Raptor and ULK1 phosphorylation; this autophagy acts as a survival mechanism, and autophagy inhibition enhances adenosine-induced CCA cell death	Lertsuwan et al. (2021)
Atractylodin (ATD)	Plant-derived polyacetylene compound from Atractylodes lancea	*In vitro* (HuCCT-1 cells)	Inhibits PI3K/AKT/mTOR and p38MAPK signaling, induces Beclin-1- and LC3-associated autophagy, and suppresses CCA cell proliferation, migration, and invasion	Acharya et al. (2021)
Derrischalcone (DC)	Plant-derived chalcone isolated from Derris indica fruit	*In vitro* (KKU-M156 and KKU-100 cells)	Induces ROS-mediated mitochondrial apoptosis via upregulation of Bax and cytochrome c, and suppresses Akt/mTOR/cyclin D1 and FAK signaling to inhibit proliferation, colony formation, migration, and invasion	Wandee et al. (2021)
Hypocrellin A (HA)	Natural small molecule isolated from Shiraia bambusicola/Shiraia bambusae	*In vitro* and *in vivo* (RBE and HuccT1 cells; HuccT1 xenograft and KRAS/P19/SB primary ICC models)	Suppresses PI3K/AKT/mTOR, MAPK, and STAT3 signaling by inhibiting phosphorylation of key pathway proteins, thereby reducing ICC cell proliferation, migration, and invasion and promoting apoptosis	Chen et al. (2024)
Chlorella sp. ethanolic extract	Microalga-derived ethanolic extract from Chlorella sp.	*In vitro* and 3D spheroid model (KKU055, KKU100, KKU213A cells)	Suppresses AKT/mTOR signaling and downregulates Bcl-2, thereby activating caspase-dependent apoptosis in CCA cells	Sawasdee et al. (2023)
Baicalin	Plant-derived flavone compound from Scutellaria baicalensis	*In vitro* (QBC939 cells)	Activates AMPK-mediated raptor phosphorylation and suppresses the mTORC1/p70S6K axis, thereby inducing caspase-3/PARP-associated apoptosis in CCA cells	Jia et al. (2022)

### mTOR signaling in chemoresistance and therapeutic sensitivity

Chemotherapy remains an important treatment option for patients with advanced or unresectable CCA, with commonly used agents including gemcitabine (GEM), 5-fluorouracil, and cisplatin (Elvevi et al., 2022). However, as tumor cells adapt to chemotherapeutic stress, drug efficacy often gradually declines (Marin et al., 2018). Although GEM-based combination regimens can improve treatment responses to some extent, their clinical application is frequently accompanied by adverse effects such as hepatotoxicity, immunosuppression, and peripheral neuropathy (Hryciuk et al., 2018). In addition, the development of chemoresistance remains a major factor limiting therapeutic efficacy and adversely affecting patient prognosis in CCA (Kannampuz et al., 2023).

Aberrant mTOR signaling represents a primary molecular mechanism driving chemoresistance in CCA. This pathway can regulate apoptosis and autophagy and interact with multiple resistance-associated signaling networks, thereby influencing tumor cell responses to chemotherapeutic agents. Emerging evidence suggests that targeted modulation of mTOR signaling may partially reverse resistant phenotypes in CCA. In terms of apoptosis regulation, blockade of AKT/mTOR signaling promotes PARP cleavage and activates apoptotic programs, thereby enhancing the antitumor effects of gemcitabine and cisplatin in CCA cells (Jang et al., 2020). Similarly, miR-199a-3p can increase cisplatin sensitivity by suppressing mTOR signaling and downregulating MDR1 expression (Li et al., 2017).

Autophagy is another important intermediate process through which mTOR regulates chemosensitivity in CCA. In iCCA specifically, highly expressed UCK2 activates the PI3K/AKT/mTOR pathway. This activation suppresses autophagy, attenuates cisplatin-induced DNA damage, and promotes the development of cisplatin resistance. Inhibition of UCK2 or combined blockade of mTOR signaling restores autophagic activity and enhances cisplatin sensitivity (Wu et al., 2024). These findings suggest that the effect of mTOR on drug response is not limited to apoptosis regulation but also involves autophagy-dependent modulation of DNA damage and cellular stress responses.

In addition to apoptosis and autophagy, mTOR signaling can influence chemotherapeutic responses through metabolic regulation, oxidative stress, receptor tyrosine kinase signaling, and inflammation-related pathways. Metformin enhances the antitumor effect of cisplatin. It achieves this by activating AMPK, inhibiting AKT/mTOR signaling, modulating the ROS/Nrf2 pathway, and promoting mitochondria-dependent apoptosis (Wandee et al., 2018). FGFR2 inhibition can also increase gemcitabine sensitivity in CCA cells by blocking the FGFR2–AKT/mTOR signaling axis (Jaidee et al., 2022). In gemcitabine-resistant CCA cells, KIF18A silencing suppresses both the PI3K/AKT/mTOR and NF-κB pathways. It downregulates Bcl-2, and increases cleaved PARP, thereby inducing apoptosis and reducing the proliferation, migration, and invasion of resistant cells. However, this study did not observe a significant enhancement of gemcitabine sensitivity following KIF18A silencing (Tanasuka et al., 2025). This distinction is important, as it indicates that suppression of resistance-associated malignant phenotypes does not necessarily translate into restored chemosensitivity.

Notably, the influence of mTOR-related signaling on therapeutic response in CCA is not restricted to conventional chemotherapy (Jaidee et al., 2022). With increasing investigation of targeted therapies against FGFR, EGFR, SRC, and other oncogenic drivers, the relationship between mTOR downstream signaling, bypass activation, and acquired resistance has attracted growing attention. In FGFR2-driven CCA and related tumor models, selective FGFR inhibition can be followed by diverse resistance mechanisms, providing a rationale for subsequent combination or sequential therapeutic strategies (Facchinetti et al., 2024). FGF10/FGFR2 signaling promotes migration and invasion of ligand-responsive CCA cells, whereas FGFR inhibition attenuates FGF10-mediated cell migration, further supporting the FGFR2 axis as an actionable target in CCA (Oeurn et al., 2023).

In FGFR2 fusion-positive CCA, resistance to FGFR inhibitors can be accompanied by upregulation of PI3K/AKT/mTOR signaling, and combined mTOR inhibition may partially restore sensitivity to FGFR inhibition in resistant cells (Krook et al., 2020). In addition, EGFR feedback activation can sustain MEK/ERK and mTOR-related downstream survival signaling, thereby weakening the efficacy of FGFR inhibitors. Combined EGFR inhibition may help achieve more durable suppression of downstream survival pathways and enhance the response to FGFR-targeted therapy (Wu et al., 2022). Furthermore, FGFR and VEGFR signaling may influence lymphangiogenesis and immune escape through HK2-related metabolic remodeling and the PI3K/AKT/mTOR–HIF-1α axis, suggesting that dual FGFR/VEGFR inhibition may provide an additional strategy for combination targeted therapy in intrahepatic CCA (Peng et al., 2023). In IDH-mutant intrahepatic CCA, the SRC/S6K–S6 axis may regulate targeted therapy sensitivity in a manner that is relatively independent of canonical upstream mTOR regulation (Luk et al., 2024), details in Table 3.

**TABLE 3:** mTOR-related therapeutic sensitivity and resistance mechanisms in cholangiocarcinoma.

Category	Interventions	Mechanisms	Key findings	Ref.
mTOR-mediated chemosensitization	RAD001+ gemcitabine	Suppression of mTOR downstream signaling with activation of apoptotic pathways and inhibition of choline kinase activity	Synergistically inhibits CCA growth by enhancing death receptor- and mitochondrial apoptosis-related signaling and altering choline metabolism.	Li et al. (2018)
	GDC-0980+ gemcitabine and/or cisplatin	Dual inhibition of PI3K and mTOR signaling with enhanced apoptotic response	Enhances the antiproliferative effects of gemcitabine and cisplatin in CCA cells by reducing AKT, mTOR and S6K1 phosphorylation, increasing PARP cleavage, and suppressing xenograft tumor growth.	Jang et al. (2020)
	UCK2 inhibition + cisplatin	Suppression of UCK2-driven PI3K/AKT/mTOR activation and restoration of autophagy	UCK2 promotes iCCA progression and cisplatin resistance by activating PI3K/AKT/mTOR signaling, inhibiting autophagy, and decreasing DNA damage response; UCK2 knockdown sensitizes iCCA cells to cisplatin.	Wu et al. (2024)
	miR-199a-3p overexpression + cisplatin	Inhibition of mTOR signaling and MDR1 expression	miR-199a-3p enhances cisplatin sensitivity in CCA cells by directly targeting mTOR, reducing phosphorylation of 4EBP1 and p70S6K, and decreasing cisplatin-induced MDR1 expression.	Li et al. (2017)
	Metformin + cisplatin	Activation of AMPK with suppression of Akt, mTOR and p70S6K signaling	Metformin enhances cisplatin-induced antiproliferation and apoptosis in CCA cells, induces S-phase arrest through p53/p21 signaling, and suppresses migration and invasion partly by reducing FAK activation.	Wandee et al. (2018)
	Metformin + cisplatin	Induction of ROS and GSH redox stress with suppression of Nrf2-mediated antioxidant response	Metformin enhances cisplatin-induced cytotoxicity and apoptosis in CCA cells by increasing ROS formation, disrupting GSH redox balance, suppressing Nrf2 and HO-1 expression, and triggering mitochondrial dysfunction through loss of mitochondrial membrane potential.	Wandee et al. (2019)
	Metformin + sorafenib/5-fluorouracil/As2O3	Activation of AMPK with inhibition of mTOR/HIF-1α/MRP1 signaling and ERK phosphorylation	Metformin suppresses ICC cell proliferation by inducing apoptosis, G0/G1 arrest and colony formation inhibition, and enhances sensitivity to sorafenib, 5-fluorouracil and As2O3, but not significantly to gemcitabine.	Ling et al. (2014)
FGFR/mTOR-targeted resistance reversal	Infigratinib + gemcitabine	Inhibition of FGFR2-driven AKT/mTOR signaling and EMT-associated pathways	FGFR2 inhibition enhances gemcitabine-mediated suppression of CCA cell growth, migration and invasion by reducing AKT/mTOR and STAT3 signaling, downregulating vimentin and slug, and suppressing angiogenesis-related VEGF expression.	Jaidee et al. (2022)
	Infigratinib + PKI-402	Concurrent inhibition of FGFR and PI3K/mTOR signaling with induction of autophagic cell death	Dual blockade synergistically suppresses CCA cell viability, induces G2/M cell cycle arrest and apoptosis, and promotes autophagic cell death by reducing p-mTOR and increasing LC3B-II accumulation.	Mahaamnad et al. (2024)
	FGFR inhibitors + INK128/sapanisertib	Suppression of PI3K/AKT/mTOR-mediated bypass signaling in FGFR inhibitor-resistant cells	Acquired FGFR2 p.E565A and p.L617M mutations reduce sensitivity to infigratinib and other FGFR inhibitors. PI3K/AKT/mTOR signaling is upregulated in resistant cells, and mTOR inhibition with INK128 synergistically resensitizes resistant cells to FGFR inhibition.	Mahaamnad et al. (2024)
	infigratinib or pemigatinib + afatinib/gefitinib	Blockade of EGFR-mediated adaptive feedback signaling with durable suppression of MEK/ERK and mTOR signaling	EGFR feedback activation limits FGFR inhibitor efficacy and contributes to resistance in FGFR2 fusion-positive CCA. Combined FGFR and EGFR inhibition durably suppresses MEK/ERK and mTOR signaling, increases apoptotic cell death, and induces marked tumor regression in patient-derived models.	Wu et al. (2022)
SRC/S6K-S6-targeted growth suppression	Dasatinib ± M2698	SRC inhibition activates MAGI1-PP2A, causing mTOR-independent S6K/S6 dephosphorylation	IDH-mutant ICC cells are hypersensitive to dasatinib; M2698 further suppresses pS6 and enhances growth inhibition in organoid and PDX models.	Luk et al. (2024)

Overall, mTOR-related signaling contributes not only to chemoresistance and therapeutic sensitivity in CCA but also to targeted therapy response, bypass pathway activation, and acquired resistance. Through extensive crosstalk with FGFR, EGFR, PI3K/AKT, AMPK, NF-κB, SRC, and S6K/S6 signaling networks, mTOR acts as a central adaptive node that shapes treatment response and provides a mechanistic rationale for combination therapy strategies.

## Discussion

The evidence synthesized in this review highlights the mTOR pathway as a critical nexus linking oncogenic activation, metabolic adaptation, and malignant progression in CCA. Rather than acting merely as a bystander, this signaling axis fundamentally shapes therapeutic response and the evolution of drug resistance. Therefore, mechanistic and therapeutic investigations centered on the mTOR signaling axis may provide important insights into CCA progression from both tumor biological and precision treatment perspectives.

Although increasing evidence has linked mTOR-related signaling to therapeutic response, drug resistance, and combination treatment strategies in CCA, its clinical translation remains at an early exploratory stage. Numerous scientific groups, particularly in mainland China and Taiwan, have published extensively on the inhibition of the PI3K/AKT/mTOR pathway, contributing to an already abundant literature of biochemical details (Liang et al., 2024; Wu et al., 2019; Peng et al., 2022). However, while the preclinical mechanisms of this important metabolic pathway have been comprehensively described, we must acknowledge that the conclusion for current clinical practice is rather disappointing (Sun, 2021). An abbreviated phase II study of single-agent MK-2206 in eight previously treated patients with advanced biliary cancer reported no objective responses, although two patients achieved stable disease for more than 12 weeks (Ahn et al., 2015). For instance, a phase 2 study utilizing the phosphatidylinositol 3-kinase inhibitor copanlisib in combination with gemcitabine and cisplatin in advanced biliary tract cancer highlighted the immense challenges of translating these molecular targets into robust survival benefits (Sun, 2021). Due to these translational hurdles, clinically meaningful evidence mainly comes from case reports or specific therapeutic contexts involving defined molecular backgrounds. For example, a patient with advanced intrahepatic CCA harboring a PIK3CA mutation was reported to achieve tumor shrinkage and maintain a partial response for approximately 6.5 months after everolimus treatment (Bian et al., 2017). Another patient with metastatic CCA carrying alterations in TSC1, TSC2, and ARID1A also achieved prolonged disease control following mTOR-related therapy (Daugan et al., 2024). In the context of liver transplantation-related management, mTOR inhibitor-based immunosuppressive regimens have also been explored in postoperative management for patients with perihilar and intrahepatic CCA; however, their association with overall survival or recurrence-free survival has not been consistently validated (Semaan et al., 2025). These clinical observations suggest that mTOR-directed interventions have shown preliminary therapeutic signals in CCA, but their clinical value still requires further validation according to molecular background, disease stage, and treatment context.

The present and future of precision oncology and tumor-agnostic therapeutic approaches for CCA have become a dominant theme in the treatment of biliary tract cancers (BTCs) (Karasic et al., 2023; Scott et al., 2022; Wang and Wang, 2023; Tomczak et al., 2022; Farha et al., 2023; Yao et al., 2023). Subtypes of BTC correspond to distinct molecular characteristics, making it a molecularly heterogenous collection of tumors. Classification based on pathological subtypes provides a useful framework for understanding this heterogeneity (Cowzer and Harding, 2022). Indeed, these subtypes exhibit distinct clinical outcomes and molecular features (Spencer and King, 2025). Metabolic heterogeneity also appears to vary according to the anatomical origin of BTC, as differential FABP5 and PGC-1 expression suggests distinct fatty acid-associated energy metabolism programs among BTC subtypes (Nakagawa et al., 2020). However, updated data on gene mutations associated with CCA reveal a complex genetic landscape with diverse targetable alterations, such as FGFR2 (4%–9%) and IDH1 (3%–14%) (Esmail et al., 2024). These findings indicate that classification based solely on anatomical location or conventional pathological features is no longer sufficient to meet the needs of precision therapy (Limaye et al., 2026; Mody et al., 2022; Lin et al., 2021; Andraus et al., 2023). As an excellent example of this molecular stratification, Montal et al. performed an integrated multi-platform analysis of 189 extrahepatic CCA samples using whole-genome expression profiling, targeted DNA sequencing, and immunohistochemistry. They found that KRAS, TP53, ARID1A, and SMAD4 were among the most frequently mutated genes, and approximately 25% of tumors harbored potentially actionable genomic alterations. Based on these data, extrahepatic CCA was classified into four molecular subtypes: metabolic, proliferation, mesenchymal, and immune. Notably, the proliferation subtype was characterized by enrichment of MYC target genes, ERBB2 alterations, and activation of the mTOR signaling pathway, with mTOR and CDK4/6 inhibitors proposed as potential therapeutic strategies for this subgroup (Montal et al., 2020).

Earlier studies have suggested that ARID1A mutations may be closely associated with aberrant activation of the PI3K/AKT pathway (Tomczak et al., 2022; Farha et al., 2023). Tessiri et al. analyzed cBioPortal data from 795 CCA samples and found that ARID1A alterations co-occurred with alterations in EPHA2, PIK3CA, and LAMA1. Among these, PIK3CA is an important component of the PI3K/AKT pathway. Further *in vitro* experiments showed that ARID1A-deficient CCA cells were more sensitive to the AKT inhibitor MK-2206 (Yao et al., 2023). However, this evidence was mainly derived from public database analyses and *in vitro* cell experiments, and still lacks validation in animal models and prospective clinical studies. Therefore, whether ARID1A deficiency can serve as a predictive biomarker for PI3K/AKT/mTOR-targeted therapy in CCA remains to be further determined.

Combination therapy targeting the mTOR signaling pathway may help overcome the limited efficacy and frequent resistance associated with single-agent treatment. However, current combination strategies still face two major challenges. First, under sustained therapeutic pressure, tumor cells may develop new adaptive resistance mechanisms through EGFR feedback activation, reactivation of PI3K/AKT/mTOR signaling, compensatory enhancement of the SRC/S6K–S6 axis, or metabolic remodeling. These mechanisms may limit the long-term efficacy of combination therapy. Second, combined use of chemotherapy, targeted agents, and mTOR inhibitors may increase hepatotoxicity, immunosuppression, peripheral neuropathy, and other systemic adverse effects, thereby affecting patient tolerance and treatment continuity. Therefore, future studies should shift from simply pursuing broader pathway inhibition toward more precise combinatorial modulation. It will be necessary to integrate molecular subtyping of CCA, identify mTOR-related resistance feedback loops, and screen predictive biomarkers for patients most likely to benefit from combination therapy. At the same time, optimization of drug dosage, treatment sequence, and therapeutic windows will be essential to enhance antitumor synergy while minimizing cumulative toxicity.

In addition to classical targeted agents, studies on traditional Chinese medicine-derived compounds and compound formulations provide complementary evidence for targeting mTOR-related pathways. Tanshinone IIA, one of the major active components of Salvia miltiorrhiza, has shown antitumor activity in preclinical studies across multiple cancer types (Ansari et al., 2021). In CCA, Tanshinone IIA may exert anticancer effects by inhibiting the PI3K/AKT/mTOR pathway (Liu et al., 2021). In intrahepatic CCA, the traditional Chinese medicine formulation Biejia-Ruangan tablet may exert antitumor effects not only by acting directly on tumor cells but also regulate cancer-associated fibroblasts through the HIPPO–PI3K/AKT signaling cascade and affect tumor microenvironmental features such as fibrosis, hypoxia, angiogenesis, and immune infiltration (Xu et al., 2025). Therefore, traditional Chinese medicine-derived compounds and compound formulations may serve as complementary directions for exploring mTOR-related combination therapy. Nevertheless, their successful clinical translation requires rigorous studies to resolve key issues, particularly the lack of robust clinical validation, unclear pharmacokinetics, possible toxicity, and the urgent need for standardized active components.

Collectively, mTOR-related signaling represents a central regulatory network linking oncogenic activation, metabolic adaptation, malignant phenotypes, therapeutic response, and drug resistance in CCA. Although preclinical and early clinical evidence supports the therapeutic potential of targeting this pathway, future studies should focus on molecularly stratified patient selection, rational combination strategies, resistance monitoring, and toxicity management. Such efforts may help transform mTOR-centered therapeutic concepts into more precise and clinically applicable strategies for CCA.

## Conclusion

In summary, aberrant mTOR signaling acts as a central driver of disease progression and therapeutic resistance in CCA, presenting a promising vulnerability for molecular subtype-guided precision oncology and rational combination therapies. Although existing studies have shown exploratory value in specific molecular contexts and selected combination treatment settings, current clinical evidence is still mainly derived from case reports, retrospective analyses, and early-stage studies. Therefore, the available evidence remains insufficient to support the widespread clinical application of mTOR-targeted strategies in CCA. To overcome these translational barriers, future research must clearly prioritize the following key directions: (Razumilava and Gores, 2014): biomarker-driven patient selection to identify individuals most likely to benefit from mTOR-directed therapies (Elvevi et al., 2022); elucidation of adaptive resistance mechanisms and mTOR-related feedback loops; and (Valle et al., 2016) systematic optimization of the dosage, sequencing, and therapeutic windows for rational combination regimens. In addition, traditional Chinese medicine-derived compounds and compound formulations may represent a complementary approach for targeting mTOR-related pathways. However, their actual therapeutic value remains to be further evaluated, with future studies needing to address the lack of clinical validation, clarify their pharmacokinetics, assess potential toxicity, and establish standardized active components.
